# Real-world use of pegylated erythropoietin in CKD-related anemia: an Indian modified Delphi consensus

**DOI:** 10.3389/fneph.2026.1864394

**Published:** 2026-09-08

**Authors:** Vijay Kher, Jatin Kothari, Jitendra Kumar, Sanjay Srinivasa, Prashant Kedlaya, Sivalingam M, Rammohan Bhat, Rajesh Nair, Mallikarjuna HM, Reetesh Sharma, Vijay Kumar Sinha, Sreedhara C. G., Jay Kumar Sharma, Iain C. Macdougall

**Affiliations:** 1 Chairman, Nephrology, Epitome Kidney Urology Institute & Lions Hospital B-Block, Khizarabad Bhagol, New Friends Colony, New Delhi, India; 2 Principal Director of Nephrology, Chief Consultant- Kidney Transplantation, Nanavati Max Hospital, Mumbai, Maharashtra, India; 3 Chairman, Department of Renal Sciences & Transplant Medicine, Accord Superspeciality Hospital, Budena Village, Sector 86, Faridabad, Haryana, India; 4 Dr. Sanjay ‘s Center for Kidney and Diabetes, Yelahanka, Bengaluru, Karnataka, India; 5 Professor and Head, Dept of Nephrology, St Johns Medical College Hospital, Bengaluru, India; 6 HOD, Department of Nephrology, Sundaram Medical Foundation (SMF), Chennai, India; 7 Director, Institute of Renal Sciences - Kauvery Hospital, Bangalore, India; 8 Prof and Head, Department of Nephrology and Kidney Transplantation, Amrita Institute of Medical Sciences, Kochi, Kerala, India; 9 Consultant Nephrologist & Transplant physician, Sagar Hospitals DSI, Bengaluru, India; 10 Chairman, Nephrology & Kidney Transplant, Yatharth Superspeciality Hospital, Sector 20, Faridabad, India; 11 Senior Director, Nephrology & Kidney Transplant, Max Superspeciality Hospital, Noida Sector 128, Noida, India; 12 Consultant Nephrologist, Institute of Nephro-Urology, Bengaluru, India; 13 Medical Affairs, Intas Pharmaceuticals Limited, Corporate House, S. G. Highway, Thaltej, Ahmedabad, Gujarat, India; 14 Professor (Retired), Clinical Nephrology, King’s College Hospital, London, United Kingdom

**Keywords:** anemia, chronic kidney disease, erythropoiesis-stimulating agents, expert consensus, modified Delphi consensus, PEGylated erythropoietin

## Abstract

Anemia is a common and clinically significant complication of chronic kidney disease (CKD), contributing to impaired quality of life, increased cardiovascular risk, and higher mortality. Although erythropoiesis-stimulating agents (ESAs) remain the cornerstone of anemia management, conventional short- and intermediate-acting ESAs are associated with frequent dosing requirements, treatment burden, and variability in haemoglobin (Hb) control. Methoxy polyethylene glycol epoetin beta (MPG-EPO; PegEPO), a long-acting ESA with an extended half-life, enables less frequent administration and may offer advantages in Hb stabilization, treatment adherence, and patient convenience. Despite evidence supporting its efficacy and safety, the utilization of PegEPO in India remains limited. To provide practical guidance for the use of PegEPO in Indian clinical practice, a panel of 12 experienced Indian nephrologists participated in a three round modified Delphi consensus process. A comprehensive review of the available literature informed the development of consensus statements covering key domains, including patient selection, treatment initiation and dose adjustment, iron management, conversion from other ESAs, Hb targets and monitoring, safety considerations, and economic aspects of therapy. Consensus was achieved through iterative rounds of expert evaluation and discussion. The expert panel agreed that PegEPO offers efficacy and safety comparable to conventional ESAs in patients with CKD-associated anemia while providing additional advantages such as reduced dosing frequency, improved Hb stability, and the potential to enhance treatment adherence. The panel also identified practical considerations for optimizing PegEPO use in both dialysis-dependent and non-dialysis-dependent CKD populations within the Indian healthcare setting. This modified Delphi consensus provides evidence-informed, expert-driven recommendations to support individualized and pragmatic use of PegEPO in routine clinical practice. However, these statements represent expert consensus and are not intended as clinical guidelines; therefore, they should be adapted based on individual patient context and local clinical practice.

## Graphical Abstract

**Figure:**
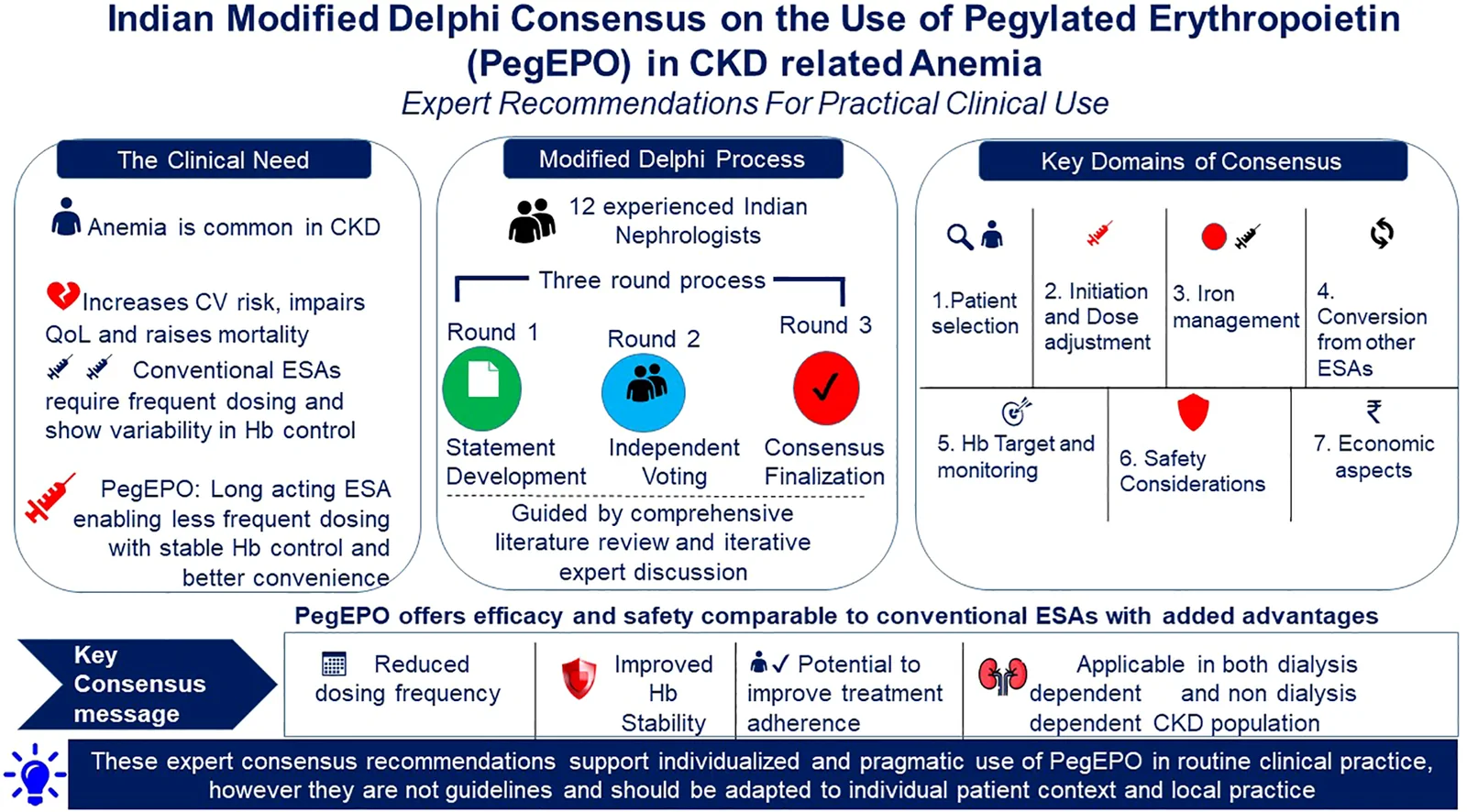

## 1 Introduction

Chronic kidney disease (CKD) remains one of the global health concerns and has been identified as a significant risk factor for the development of end-stage renal disease and cardiovascular morbidity and mortality (1). Anemia is one of the frequent manifestations of CKD and largely results from inadequate erythropoietin production, iron deficiency and inflammation, therefore associated with reduced exercise capacity, impaired quality of life (QOL), faster CKD progression, higher hospitalisation and mortality (2). In India and other Asian regions, the prevalence of renal anemia remains particularly high (40-70%) and steadily increases with stages of severity of CKD (3, 4).

Erythropoiesis-stimulating agents (ESAs) remain the cornerstone pharmacotherapy for CKD-induced anemia for more than two decades after correcting underlying reversible causes like iron deficiency anemia and inflammation (5). Recent Kidney Disease: Improving Global Outcomes (KDIGO) 2026 anemia guidelines, as well as Consensus guidelines published by the Indian Society of Nephrology, recommend judicious use of ESAs to keep hemoglobin levels within a ‘conservative’ target range, taking into account both symptomatic relief and potential risks of excess hemoglobin levels, as well as excessive doses of ESAs (4, 6). Conventional ESAs, such as darbepoetin alpha and epoetin alfa, must be administered on a weekly or every two-week basis, are prone to hemoglobin cycling, demand a significant amount of clinic time, and place a significant treatment load on both CKD patients and dialysis centre staff (1, 7, 8).

Methoxy polyethylene glycol epoetin beta (pegylated erythropoietin; PegEPO, continuous erythropoietin receptor activator) is a ‘third generation’ ESA formed by covalent attachment of a methoxy-polyethylene glycol chain to the epoetin beta molecule, thereby enormously increasing its half-life and the duration of erythropoietin receptor activation (1). Its long half-life of about 130 hours for dialysis and non-dialysis CKD patients, given either subcutaneously and/or intravenously, allows its once-monthly maintenance doses to provide more stable levels of hemoglobin (9). Phase III studies and post-marketing experience have proved PegEPO to be as effective and well-tolerated as conventional ESAs for both correcting and maintaining renal anemia. Additionally, it also offers longer-dosing intervals and possibly lowers healthcare system load (4). Real-world studies conducted on Indian and Asian patients with CKD have indicated that effective dose schedules of PegEPO are capable of improving hemoglobin levels (10, 11). In a multi-centre observational study conducted among Indian patients (HEMEPEG), significant increase in hemoglobin levels (10– 12 g/dl) in about 50% of patients were achieved with weekly or 10-day treatment with 30 µg doses of PegEPO (11). One of the important causes for anemia in patients with CKD, particularly in dialysis, is erythropoietin resistance. In patients undergoing treatment with erythropoietin, 10% become resistant and require high doses of the drug (12); higher doses are associated with increased morbidity and mortality (13). Treatment with PegEPO in patients with CKD resulted in a hemoglobin increase comparable to darbepoetin, demonstrating the need to reduce the therapeutic dose (14).

Although pegylated erythropoietin (PegEPO) and its biosimilar (Erypeg; Intas Pharmaceuticals) approved by the DCGI in 2015 (15), have been available in India for several years for the treatment of CKD-associated anemia, their adoption in clinical practice remains suboptimal. Data from the DOPPS study also indicate that PegEPO is prescribed in only 3–26% of patients despite its established efficacy, favorable pharmacokinetic profile, and reduced administration burden (16). Recognizing this evidence–practice gap, a modified Delphi expert consensus was conducted to develop context-specific recommendations for the use of PegEPO in India, with the goal of optimizing anemia management and improving clinical outcomes in patients with CKD.

## 2 Methodology

### 2.1 Selection of panel and development of the expert panel questionnaire

For the modified Delphi consensus, an expert panel comprising 12 clinicians was convened. All panelists were qualified nephrologists with more than 15 years of clinical experience in the management of renal anemia across hospital-based and independent clinical settings.

Experts were purposively selected using predefined eligibility criteria, including (i) substantial clinical experience in chronic kidney disease (CKD) anemia management and (ii) active involvement in nephrology practice. Potential participants were identified through established professional networks.

Eligible experts were invited via email, with information provided regarding the study objectives and Delphi process. All invited experts agreed to participate, and no eligible invitees declined participation. The final panel included geographically diverse representation: 7 experts from southern India, 4 from northern India, and 1 from western India.

To ensure methodological rigor and minimize bias, a steering committee comprising three senior nephrologists independent of the Delphi panel was constituted to oversee study design, questionnaire development, and interpretation of results. Additional support was provided by other nephrology experts where required.

The Delphi process consisted of three iterative rounds. All 12 panelists completed all rounds, with no attrition reported. Data from relevant studies evaluating the dosing, efficacy, and safety of PegEPO were shared with the expert panel for review as part of the preparatory phase for the consensus development. Key topics requiring clinical expertise were identified, discussed, and finalised by the panel chair. These topics included patient selection, dosing strategies, iron management, switching from other ESAs, target hemoglobin levels and monitoring, safety outcomes, and cost considerations. A pre-meeting reading material was distributed to all panel members to prepare for the meeting, based on these domains. This material mainly focused on data regarding PegEPO’s utility in CKD anemia. Subsequently, an advisory board meeting was conducted before the round-1 survey, during which survey-related topics were discussed in detail. Another in-person meeting was used to facilitate structured discussion and raise relevant issues for consideration. Survey questions were finalised following consultation with the panel chair.

### 2.2 Modified delphi survey

**Figure 1:**
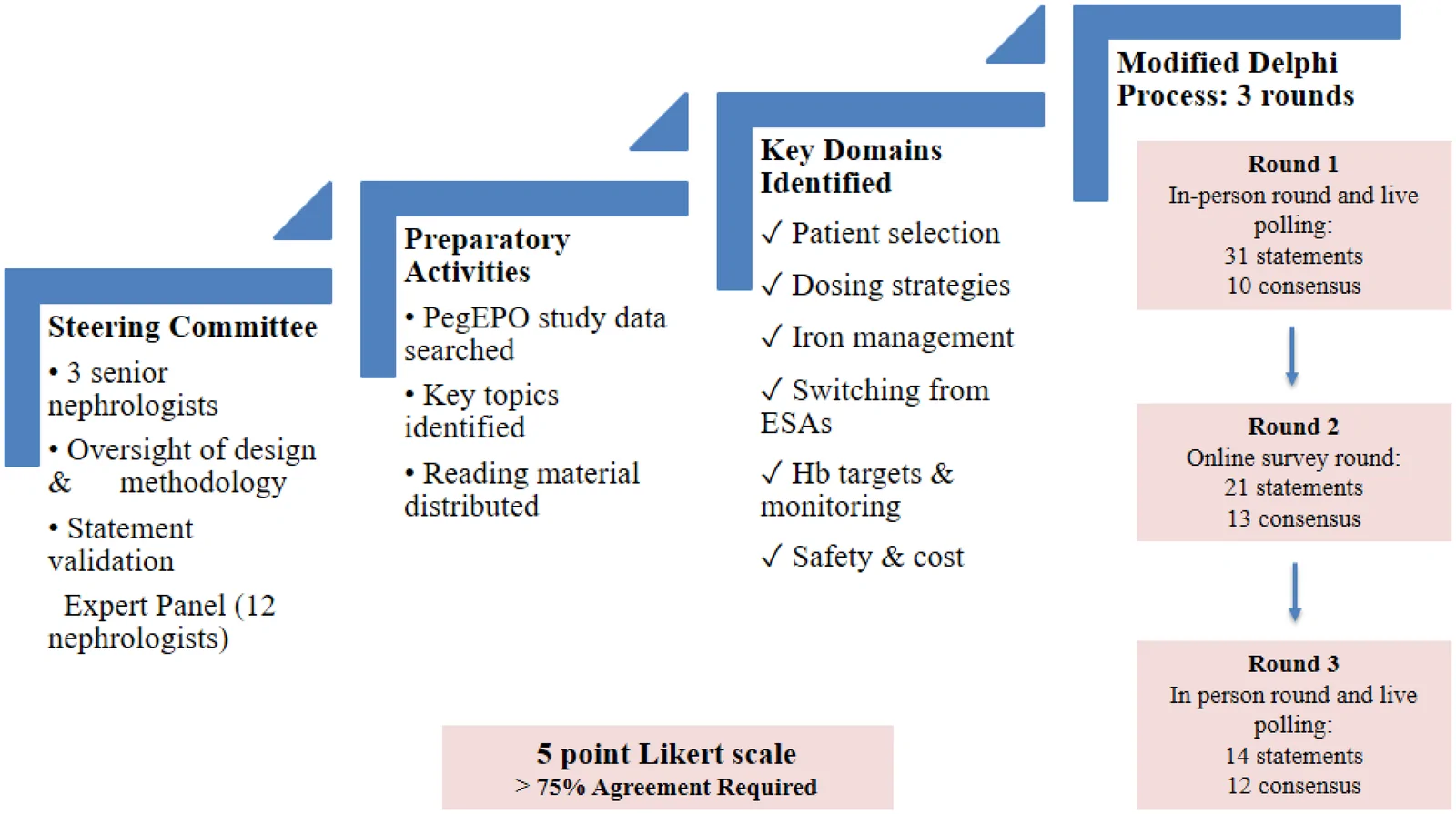
The process flow chart for consensus development.

The modified Delphi consensus on the use of PegEPO in CKD-related anemia included two in-person meetings and three rounds of online surveys. Live polling in Rounds 1 and 3 were conducted following the in-person meetings, during which panelists held in-depth discussions based on a pre-designed questionnaire assessing the utility of pegylated erythropoietin in CKD anemia. Due to this structured, face-to-face format, anonymity could not be maintained in these rounds. In contrast, Round 2 was conducted entirely online without a meeting, allowing all panelists to submit their responses independently; therefore, anonymity was preserved only in Round 2. The process flow chart is presented as **Figure 1**. Consensus for a statement was predefined as agreement by> 75% of panellists (≥10 of 12), on a 5-point Likert scale. (1-Strongly Disagree, 2-Disagree, 3-Neutral, 4-Agree, 5-Strongly Agree). This was used in all three rounds of polling. Feedback from panelists was obtained after Rounds 1 and 2 through individual telephonic discussions. A process of controlled feedback was implemented, whereby summarized responses were used to iteratively refine the questionnaire for subsequent rounds. Disagreements identified during the initial in-person meeting were systematically documented, and the corresponding statements were reintroduced in later rounds to facilitate re-evaluation and convergence toward consensus. Round-1 consisted of 31 questions for an online survey conducted between September 21 and September 30, 2025. Based on the analysis of round-1 responses, round-2 was conducted between November 15 and November 30, 2025, which included 21 close-ended online survey questions. This was followed by a second in-person meeting to discuss areas lacking consensus. Round-3 comprised 12 online survey questions.

Based on inputs provided by the advisors across three rounds, a total of 35 statements were generated. These statements were categorized by clinical setting and addressed key domains including patient selection, dosing and administration, iron management, switching from other (ESAs, hemoglobin targets and management, safety outcomes, and cost considerations (**Figure 1**).

In round-1, consensus was achieved for 10 of the 31 statements. Statements that did not reach consensus were reviewed, refined, and subsequently included in round-2, which was conducted online. Overall, consensus was achieved for 13 of 21 statements evaluated in round-2. Following the two survey rounds, an in-person advisory board meeting was conducted in December 2025, and topics that did not have consensus in two rounds were discussed. In round-3, 14 statements were subjected to live polling, adding few new questions for discussion. Consensus was achieved for 12 of these 14 statements. Collectively, a total of 35 consensus statements were finalized, and final recommendations were made.(Suppl_1) Evidence grading was assigned based on the hierarchy of available literature, including Level A: Systemic Review/Meta-analysis/Randomised trials; Level B: Prospective/Retrospective Observational evidence/Registries/Real World Evidence (Level B); Level C: National or International Guidelines/Position statements/Expert recommendations and Level D: Expert opinion/Pharmacoeconomic considerations/clinical practice-based insights where published evidence is limited (Suppl_2).

## 3 Expert opinion

### 3.1 Topic: patient selection

1. Long-acting ESA may be preferred as first choice for anemia management in NDD patients [C].

2. *De novo* patients with renal anemia can be initiated on ESA therapy using PegEPO [B].

3. PegEPO is suitable to switch patients from short-acting ESAs [A].

4. PegEPO can be considered the first-line ESA in CKD stage 5D patients [B].

5. Patients who need ESA conversion from intravenous to subcutaneous can be shifted to PegEPO [C].

6. PegEPO is safe to use in elderly CKD patients without dose adjustments beyond standard recommendation [B].

7. ESA hyporesponsive patients can be given a trial of PegEPO as ESA therapy after ruling out all the possible causes of ESA resistance [C].

8. Patients who frequently miss their ESA injections and have poor compliance can be shifted to PegEPO [B].

9. PegEPO provides better haemoglobin stabilization compared to short acting ESAs [B].

Rationale: The prevalence of anemia grows as the stage of CKD progresses from 40% in stage 3 to 70% in stage 5 (17). Due to high inflammation and functional iron deficiency, symptoms of anemia are more severe in dialysis patients than non-dialysis patients. The prevalence of anemia in stage 3a-5 CKD is 39.6% according to a study conducted by Minutolo et al. (18). However, as per the CKDoPPS study, 68% of NDD patients received neither Iron nor ESA for their anemia management (19). Furthermore, patients with stage 4 and stage 5 CKD appear to be undertreated, where only 24% and 32% of individuals receive any form of treatment, respectively (20). The majority of NDD patients suffer from mild to moderate anemia (21). A discrete choice experiment carried out with NDD CKD anemic patients found that those with anemia regard ‘the ‘method’ and ‘frequency of treatment administration’ as a significant factor (22). Longer-acting ESAs administered weekly or fortnightly are a preferable choice compared to three times short-acting ESAs, particularly in non-dialysis patients.

PegEPO has shown its efficacy in *de novo* patients in multiple clinical trials. In the CORDATUS trial, the hemoglobin response rate with monthly injection was 94.1%, which was significantly higher than the protocol-specified 60% and was comparable to Darbepoetin. It was also non-inferior to Darbepoetin in hemoglobin rise at 28 weeks. Safety profile was also similar in both the arms (23). In the ARCTOS trial, hemoglobin response rates with PegEPO were similar to Darbepoetin in *de novo* NDD patients. PegEPO injection once every 2 weeks was as effective as darbepoetin alfa once weekly for correcting anemia (24).

PegEPO has been tested in multiple trials when patients stable on other ESAs were switched to PegEPO therapy. In phase III STRIATA trial, efficacy and safety of intravenous PegEPO administered once every 2 weeks (Q2W) was evaluated for hemoglobin maintenance following direct conversion from darbepoetin alfa (DA) (25). The mean difference in the primary endpoint was clinically non-inferior to DA, and both therapies were well tolerated. The MAXIMA and PROTOS studies evaluated the efficacy of PegEPO administered via intravenous and subcutaneous routes, respectively, in maintaining hemoglobin levels in hemodialysis patients previously treated with epoetin (26, 27). In MAXIMA, 673 patients receiving intravenous epoetin once or three times weekly were randomized to continue epoetin or to switch to intravenous PegEPO administered every 2 or 4 weeks. Similarly, the PROTOS study randomized 572 patients receiving subcutaneous epoetin to continue epoetin or to receive subcutaneous PegEPO every 2 or 4 weeks. Both studies demonstrated that PegEPO, administered once monthly, maintained hemoglobin concentrations within the target range with efficacy comparable to conventional epoetin therapy, supporting its effectiveness in the management of anemia in chronic hemodialysis patients (26, 27). In a real-world study, 127 DD patients receiving conventional ESAs were switched to a once-monthly PegEPO regimen. Monthly administration of PegEPO maintained stable hemoglobin concentrations with an acceptable safety profile. A median monthly dose reduction was also observed, decreasing from 120 µg during the dose-titration period (DTP) to 100 µg during the efficacy evaluation period (EEP) (28).

In HD patients, vascular access allows the convenience of intravenous administration of ESAs. However, there are two choices in NDD, i.e. subcutaneous or intravenous. In non-hemodialysis patients, the subcutaneous route may be more convenient due to the absence of continuous intravenous access, ease of self-administration, lower dose requirements, fewer hospital visits, reduced dosing frequency, and overall lower treatment costs (29). Studies have also demonstrated that shifting patients from i.v. to s.c. injection results in significant cost benefit to the patients and total ESA dose reduction (29). Since PegEPO provides the convenience of fortnightly or monthly administration with comparable efficacy and safety, it can be considered as a preferred choice over other existing ESAs.

In a study conducted in a geriatric population (>65 years) suffering from chronic anemia, ESA therapy was associated with increases in hemoglobin and improvements in fatigue and QOL (30). In a recent meta-analysis, patients receiving higher doses of ESAs (75,000, 100,000, and 200,000 U/week) had progressively increased mortality risks, with hazard ratios of 1.85 (95% CI: 1.55–2.23), 1.89 (95% CI: 1.53–2.30), and 2.07 (95% CI: 1.46–2.95), respectively (31). Also, high doses of ESA independently predicted cardiovascular mortality and mortality from all causes (31). Recently published studies have shown that conversion from short half-life ESAs to once-monthly subcutaneous PegEPO in pre-dialysis CKD patients effectively maintained hemoglobin levels, with a significant reduction in dose requirements (32). In the MINERVA study, conversion from shorter-acting ESAs to PegEPO at doses lower than those recommended effectively maintained target hemoglobin levels in both non-dialysis and hemodialysis CKD patients, particularly among those previously receiving higher ESA doses (33). Therefore, in geriatric populations, switching to PegEPO might provide benefits in terms of dose reduction and reduced morbidity.

ESA hyporesponsiveness is commonly defined as failure to reach the target Hb range despite SC epoetin dose >300 IU/kg/week (450 IU/kg/week IV epoetin), or darbepoetin dose >1.5 mcg/kg/week, or equivalent dose of methoxy ethylene glycol epoetin beta following investigation and treatment of other causes (34). The prevalence varies from 5-20% in HD population (35).

**Figure 2:**
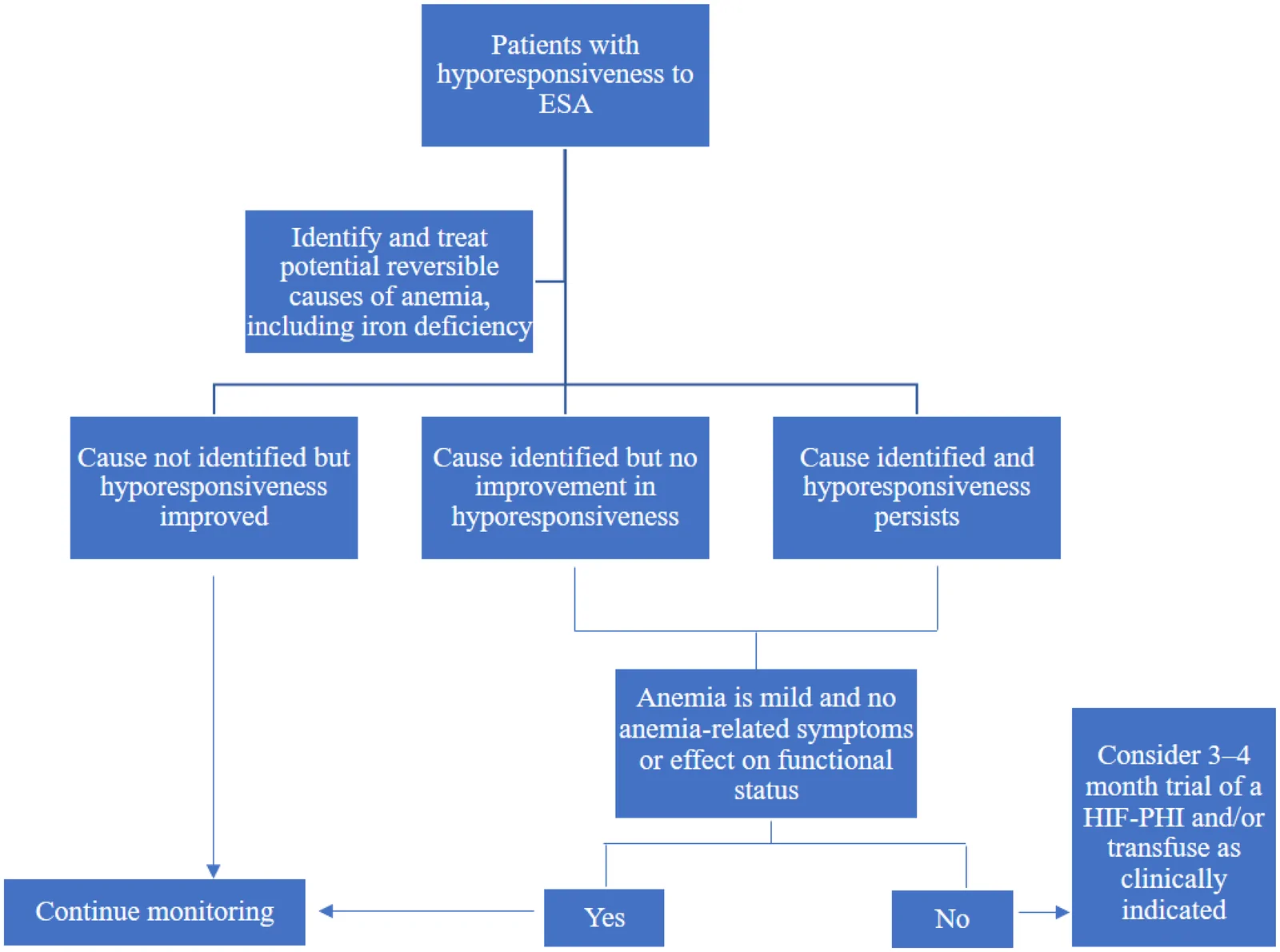
Treatment algorithm of sustained ESA hyporesponsiveness.

The most common causes of erythropoiesis-stimulating agent (ESA) hyporesponsiveness are inflammation and iron deficiency (**Table 1**). Inflammatory states impair erythropoiesis through cytokine-mediated effects on bone marrow function, reduced responsiveness to erythropoietin (EPO), and diminished endogenous EPO production. Additionally, inflammation promotes functional iron deficiency via hepcidin-mediated iron sequestration, along with other contributing mechanisms (6). Management includes identifying the causes and alternating the therapy (**Figure 2**) (6).

**Table 1:** Causes of ESA hyporesponsivesness (36).

Iron deficiency
Inflammation (infections, dialysis catheter use, and autoimmune disease)
Hyperparathyroidism
Blood loss (GI tract, dialysis procedure, and menses)
Inadequate dialysis
Malignancy
Hematologic disorders (hemoglobinopathies, multiple myeloma, hemolysis, and antibody-mediated pure red cell aplasia)
Nutritional deficiencies (copper, zinc, folate, vitamin B_12_, carnitine, and vitamin E)
Medications (RAS inhibition)
Unexplained (∼30%)

ESA hyporesponsiveness has been associated with thrombotic events in dialysis patients (36). Since increasing the doses in ESA hyporesponsive patients increases the cardiovascular morbidity, the panel agreed to switch to PegEPO to observe the response before giving supramaximal doses of short-acting ESAs, however it should only be done after ruling out all the possible causes for ESA resistance. In a study by Nand et al, Pegylated erythropoietin appeared better than darbepoetin alfa in overcoming erythropoietin hyporesponsiveness and maintaining stable hemoglobin levels in CKD patients on maintenance hemodialysis (37).

It is seen that approximately 90% of hemodialysis patients experience significant fluctuations in hemoglobin levels over time (38). Greater fluctuations in hemoglobin levels during treatment with ESAs are associated with a higher risk of infections, thrombotic complications, and all-cause mortality in patients undergoing hemodialysis (39). Studies have shown that hemoglobin variability has been associated with mortality in ND patients and is independent of the absolute hemoglobin level, change in hemoglobin level over time, and ESA use. The increased hemoglobin variability observed in patients receiving ESAs is likely multifactorial, arising from factors such as the drug’s half-life and dosing practices, fluctuations in patient clinical status, and changes in iron availability (40). ESA dosing/frequency and administration route (intravenous, subcutaneous) are important factors contributing to hemoglobin variability in patients with CKD (41). Some studies suggest that long-acting ESAs may reduce hemoglobin variability by allowing less frequent dosing and promoting more stable erythropoiesis; however, other reports show no significant difference, or even greater variability with certain agents (42). However, long-acting agents are generally presumed to maintain more stable hemoglobin levels—due to fewer dose adjustments and less frequent injections than short or intermediate-acting agents. In clinical environments characterized by high provider workload and fewer reimbursement constraints on ESA use, long-acting ESAs may be favoured in these settings. Switching to different types of ESA (e.g., longer acting ESA for noncompliant patients with frequent missing dialysis treatments) seems to be a fair option for reducing hemoglobin excursions in certain patients (41), rather than keep on increasing or decrease the dosage of the same ESA in the patient.

Newer agents like Hypoxia Inducible Factor- Prolyl Hydroxylase Inhibitors (HIF-PHIs) have evolved as an alternative to ESAs for management of renal anemia. However, they are not entirely free from limitations, including episodes of hemoglobin overshoot and variability in hemoglobin levels (43). Randomized trials and meta-analyses have consistently shown non-inferior hemoglobin efficacy between HIF-PHIs and ESAs, including long-acting agents, without clear evidence of superior hemoglobin stability in clinical practice (44, 45). Importantly, PegEPO offers well-established long-term safety data, predictable pharmacokinetics, and the advantage of once-monthly administration, which may enhance adherence and reduce treatment burden (46). While HIF-PHIs provide an alternative oral approach and may offer physiologic advantages, the overall magnitude of benefit in reducing clinically meaningful hemoglobin variability remains modest. Therefore, both therapies are effective options, with PegEPO continuing to represent a robust and reliable standard for hemoglobin control, particularly where dosing convenience, long-term experience, and predictable response are prioritised.

### 3.2 Topic: iron management

10. Ferritin >200 ng/mL and TSAT >20% should be ensured before initiating PegEPO [C].

11. Intravenous iron may be administered proactively in patients undergoing dialysis (DD) who are being initiated on PegEPO, particularly when iron deficiency is present or anticipated, to optimize hemoglobin response and improve treatment efficacy [B].

12. Patients with stable iron stores (TSAT ≥25% and ferritin ≥200 ng/mL) are more likely to have a predictable response to PegEPO [B].

13. Combining PegEPO with intravenous iron during the same visit might improve patient compliance and outcomes [B].

Rationale: Iron deficiency is one of the most important causes of ESA hyporesponsiveness (35). Therefore, guidelines suggest checking the iron stores before initiating ESA therapy (6). Iron deficiency can be either absolute or functional, therefore, correcting the body iron stores before increasing the ESA doses is a recommended step in anemia management in CKD.

Various clinical studies have supported the proactive iron dose therapy over a reactive one. In the Proactive, High-Dose versus Reactive, Low-Dose Intravenous Iron Supplementation in Hemodialysis (PIVOTAL) trial, conducted in HD patients, a proactive strategy was superior to a reactive low-dose regimen and resulted in the use of lower overall doses of ESAs (47). The median monthly dose of erythropoiesis-stimulating agent was 19.4% lower in patients receiving the high-dose regimen (47). Additionally, in this trial, the high-dose regimen of intravenous iron administered proactively resulted in a significantly lower risk of death or major nonfatal cardiovascular events as compared with that observed with a reactive, low-dose regimen. Therefore, Intravenous iron therapy may be administered proactively in dialysis-dependent patients initiating PegEPO; however, its use should be guided by iron indices, including serum ferritin and transferrin saturation (TSAT), rather than applied universally. Proactive iron supplementation is particularly beneficial in patients with absolute or functional iron deficiency to optimize erythropoietic response, improve hemoglobin stability, and reduce ESA dose requirements. At the same time, careful consideration should be given to factors such as active infection, risk of iron overload, and individual patient characteristics. Accordingly, intravenous iron administration should be individualized and based on established thresholds and clinical judgment to ensure both efficacy and safety.

Sufficient iron availability is crucial for effective erythropoiesis, as both absolute and functional iron deficiency significantly contribute to ESA hyporesponsiveness and hemoglobin variability in populations with CKD and those undergoing hemodialysis (48). KDOQI guidelines suggest that in hemodialysis patients, transferrin saturation (TSAT) should be maintained above approximately 20–25% and ferritin levels should exceed 200 ng/mL to guarantee an adequate iron supply to the bone marrow, thereby optimizing the erythropoietic response to ESA therapy (49).

As per recent KDIGO guidelines for management of CKD induced anemia, in individuals with anemia and CKD G5 undergoing hemodialysis (CKD G5HD), initiation of iron therapy is suggested when serum ferritin is ≤500 ng/mL (≤500 μg/L) and transferrin saturation (TSAT) is ≤30%. Among patients with anemia and CKD G5HD who are initiating iron therapy, intravenous (IV) iron is preferred over oral iron. In patients with CKD receiving iron therapy, it is reasonable to withhold routine iron supplementation when serum ferritin exceeds 700 ng/mL (>700 μg/L) or TSAT is ≥40% (6).

These established thresholds are indicative of targets associated with enhanced hemoglobin responses and a decrease in the need for higher ESA dosing, while lower levels of TSAT or ferritin are linked to diminished iron availability and compromised erythropoietic responsiveness.

Carelessness and insufficient disease-related information are frequently blamed for non-adherence in NDD patients (50). The presence of a central venous catheter, the actual dialysis day, procedural discomfort, the most recent advice from medical professionals, and the frequency of counselling, on the other hand, have a significant impact on compliance among DD patients (51). Perception of the importance of treatment adherence was missing in the patients in the study (51). Considering all the factors discussed, healthcare professionals should individualize interventions according to patients’ specific risk profiles, with the goal of enhancing patients’ perception of the importance of treatment adherence and combining both ESAs and Iron in a single session might improve adherence to ESA therapy. Additionally, Intravenous iron may also aid with overcoming ESA resistance that has been observed when an ESA has been used without concomitant i.v. iron (52). However, intravenous (IV) iron administration is not required at every visit for pegylated erythropoietin (PegEPO) therapy; its use is primarily guided by convenience and should be individualized based on the patient’s iron parameters.

### 3.3 Topic: switching from other ESAs

14. A once-monthly dosing frequency improves patient adherence and clinic workflow [B].

15. Longer dosing intervals with PegEPO reduce the risk of hemoglobin variability-associated adverse outcomes [B].

16. Patients with hemoglobin variability or requiring frequent dose changes on Darbepoetin/short acting ESAs can be shifted to PegEPO [B].

17. Patients with stabilized hemoglobin on fortnightly ESA injections can be considered for safely switching to monthly PegEPO without compromising anemia control [A].

18. PegEPO can maintain hemoglobin levels when administered once every 2–4 weeks, subcutaneous or intravenous, in patients who were previously maintained on EPO alfa or Darbepoetin [A].

Rationale: Dosing frequency undoubtedly has a significant impact on patients’ adherence. Reducing the frequency of dosing improved adherence to oral therapies, according to a meta-analysis (53). Once-weekly dosing was linked to better patient adherence to therapy than once-daily dosing, according to another study that examined the relationship between intermittent dosing and adherence (54). One way to increase the effectiveness of care is to use ESAs at longer dosage intervals. Once-monthly dosing reduces the amount of time needed to administer and monitor therapy and enables nephrology specialists to provide comprehensive renal care where the patient takes precedence over task-oriented procedures (55). Changes in ESA dosage or clinical events are often linked to hemoglobin variability. Compared to short-acting EPO, long-acting ESA produced greater hemoglobin stability (26). As per UKKA Clinical Practice guidelines for Anemia management in CKD, less frequent administration using long-acting ESAs may be the treatment of choice in people with CKD not on hemodialysis (34). Research has indicated that a sharp increase in hemoglobin levels is linked to poorer cardiovascular outcomes (56, 57). Within dosing intervals unique to each ESA class, the maximum efficacy of ESAs is attained (58). Epoetin alfa’s effectiveness decreases in hemodialysis patients receiving short-acting ESAs subcutaneously or intravenously when the dosage is lowered from three times weekly to once weekly, and it further declines when the interval is increased to every other week (7). On the other hand, long-acting ESAs exhibit optimal efficacy at longer, agent-specific intervals: PegEPO exhibits maximal efficacy with once-monthly (every four weeks) dosing, while darbepoetin alfa appears to be most effective when given every two weeks (59). Differences in pharmacokinetic characteristics, especially drug half-life, must be carefully taken into account when transferring patients from short-acting to long-acting ESAs (60). For example, conversion from thrice-weekly epoetin alfa to once-monthly PegEPO has been shown to substantially reduce injection frequency while maintaining hemoglobin concentrations within the recommended target range in patients with CKD. Multiple studies have confirmed that patients on stable Darbepoetin weekly or Recombinant Human erythropoietin (rHuEPO) three times a week can be converted to PegEPO monthly therapy, which results in improved anemia control, characterised by a greater increase in hemoglobin levels in patients previously treated with ESAs at extended dosing intervals. This factor should be taken into account when switching to a long-acting ESA, given its potential impact on the risk of hemoglobin overshooting. In MIRACEL study, conversion to PegEPO from Darbepoetin or short-acting ESA, was shown to be practical, convenient and offer good control of hemoglobin levels, regardless of the previous type of therapy or dosing frequency (61). Also, monthly CERA maintains hemoglobin stability in hemodialysis patients after switching from epoetin beta; however, its stronger erythropoietic stimulus may be associated with a different effect on anisocytosis (62). This difference may be attributable to higher circulating erythropoietin levels and more potent suppression of hepcidin. In a Japanese study, 61 stable patients on DA or rHuEPO were switched on 4 weekly PegEPO not only led to stable hemoglobin levels but also decrease in desired dose at 28 weeks (63). In a pooled analysis of 13 phase III trials, 2060 patients were included in the analysis. Across all analyzed subgroups, switching from shorter-acting ESAs to once-monthly continuous erythropoiesis receptor activator maintained stable hemoglobin concentrations in a high proportion of patients (78%), with only moderate hemoglobin fluctuations and a low frequency of dose adjustments (64).

### 3.4 Topic: Hb targets and monitoring

19. Hemoglobin should be monitored every 2 weeks after initiation or dose adjustment with PegEPO [C].

20. Monthly hemoglobin monitoring is sufficient after dose stabilization [C].

21. More frequent hemoglobin monitoring is required during the first two months after switching to PegEPO [C].

22. Target hemoglobin should be maintained between 10–12 g/dL in patients on PegEPO [A].

Rationale: As per guideline recommendations, hemoglobin levels should be monitored every 2–4 weeks during initiation and dose adjustment (4). Once a month hemoglobin measurement is recommended by KDIGO in the initiation phase of ESA therapy, while in maintenance therapy hemoglobin has to be measured monthly in dialysis therapy and three-monthly in non-dialysis ones (6). Also, as per UK Kidney Association guidelines, hemoglobin concentration should be monitored every 2–4 weeks during the correction phase or following any ESA dose adjustment, and subsequently every 1–3 months once stable levels are achieved during the maintenance phase of treatment (34). The upper limit of hemoglobin level for patients on ESA therapy recommended by KDIGO is 11.5 g/dl, while Indian Consensus and UKKA guidelines recommend a target of 10–12 g/dl in adults, but in no case should ESA be used intentionally to increase hemoglobin beyond 13 g/dl (4, 6, 34). Hemoglobin (Hb) targets should be individualized rather than adopting a uniform threshold for all patients. While correction of anemia is important to improve symptoms, quality of life, and reduce transfusion requirements, excessive elevation of Hb levels has been associated with increased risks, including cardiovascular events and vascular access complications. Accordingly, Hb targets should generally be maintained within a moderate range, with treatment tailored based on patient characteristics, comorbid conditions, response to therapy, and risk profile. Careful dose titration of ESAs, including PegEPO, along with regular monitoring of Hb levels, is essential to avoid rapid rises or overshooting beyond the desired range. An individualized, cautious approach helps achieve optimal balance between efficacy and safety in CKD anemia management.

The frequency of ESA dose adjustments should be guided by the rate of hemoglobin increase during the initiation phase of ESA therapy, the stability of hemoglobin levels during maintenance treatment, and the frequency of hemoglobin monitoring. In the outpatient setting, the minimum interval between ESA dose adjustments is generally two weeks, as the effects of most dose changes are unlikely to be evident over a shorter time frame (58). KDIGO recommends individualising the frequency of ESA administration based on patient preferences and type of ESA administered (6).

KDIGO also advises monitoring Hb every 2–4 weeks and adjusting the dose accordingly to avoid a rapid rise of >1.0 g/dl (>10 g/l) during that interval. To avoid a rapid decline in Hb, consider reducing the ESA dose rather than holding ESA therapy, as long as the Hb does not exceed 11.5 g/dl (115 g/l) (6).

### 3.5 Topic: dose consideration

23. The starting dose of PegEPO in *de novo* patients should be 50 µg fortnightly [D].

24. A loading dose of 100 µg can be considered in patients having hemoglobin <9 g/dl [D].

25. Fortnightly dosing is better than monthly dosing [D].

26. Conversion ratios to PegEPO are well established and practical in real-world settings [A].

27. In hemodialysis patients, PegEPO should be administered at the end of the dialysis session [C].

28. For patients not achieving the desired hemoglobin level, shortening the dosing interval is preferable to changing the dose [D].

29. In case of dose discrepancy on per kg body weight, the nearest upper µg dose can be considered for administration [D].

Rationale: For CKD patients who are not in stages III–V on dialysis or non-dialysis, the recommended starting dose is 0.6 µg/kg body weight, administered either subcutaneously or intravenously, and given once every two weeks (4). However, in a real-world scenario, converting it to a fixed µ dose for the patient remains a challenge for the physician. In India, PegEPO is available as 30, 50, 75 and 100 µg injections. In recent KDIGO guidelines, the dose proposed is 50-120 µg every two weeks or 120–200 µg every month in non-dialysis patients and 0.6 µg/kg every 2 weeks (may be rounded to the nearest upper µg dose) (6). For titration, in CKD not receiving dialysis- Increase or decrease dose and/or dosing frequency as needed (generally not given more than once every 2 weeks), while in dialysis patients- Increase by 30-50 µg/dose if hemoglobin rise is <1.0 g/dl (<10 g/l) in 4 weeks and reduce by 30–50 µg/dose if hemoglobin rise is >2 g/dl (20 g/L) in 4 weeks (6). In HEMEPEG study, 30 µg used weekly resulted in a significant rise in hemoglobin levels in DD patients (11). AFFIRM study evaluated the dose conversion ratio (DCR) in a population of hemodialysis patients who achieved comparable hemoglobin levels after switching from intravenous darbepoetin alfa to intravenous PegEPO in a real-world clinical setting (65). It concluded that European hemodialysis patients who were converted from darbepoetin alfa to PegEPO and completed 6–7 months of post-conversion PegEPO therapy, an approximately 20% increase in the microgram dose was required to maintain a comparable hemoglobin profile. The geometric mean DCR of PEG-Epo to DA was 1.17, rising to 1.21 when the effect of red blood cell (RBC) transfusions was taken into account (65). The monthly dose of PegEPO ranges from 120-360 µg/month; however individualised dosing becomes difficult with this. Studies have shown that a dose of 75-100 μg/month is enough to maintain stable levels of hemoglobin (66). In a Japanese real-world study conducted in more than 3000 patients, among patients who transitioned to PegEPO, the most frequent dose conversion patterns varied according to the prior ESA and dialysis modality (67). In those switching from recombinant human erythropoietin (rHuEPO), the most common regimens were conversion from erythropoietin <4500 IU/week to PegEPO 100 µg every 4 weeks in non-dialysis (ND) patients (22.72%), from erythropoietin ≥4500 IU/week to PegEPO 150 µg every 4 weeks in hemodialysis (HD) patients (23.04%), and from erythropoietin <4500 IU/week to PegEPO 100 µg every 4 weeks in peritoneal dialysis patients. Similarly, among patients switching from darbepoetin alfa, the most frequent conversions were from darbepoetin alfa ≥30 to <40 µg/week to PegEPO 100 µg every 4 weeks in ND patients (10.51%), from darbepoetin alfa ≥40 to <60 µg/week to PegEPO 150 µg every 4 weeks in HD patients (9.51%), and from darbepoetin alfa ≥30 to <40 µg/week to PegEPO 150 µg every 4 weeks in peritoneal dialysis patients (67).

Considering the local factors and available studies, the panel agreed that 50 µg every 15 days is the optimal dose for Indian patients, followed by fortnightly hemoglobin measurement and subsequent dose titration in the dose levels as recommended. If the patients’ hemoglobin is less than 9 g/dl, a loading dose of 100 µg can be considered and then 50 µg to be continued as recommended above. In the titration phase, hemoglobin should be measured fortnightly, followed by monthly once the patient enters the maintenance phase. Studies have shown that switching from EPO therapy to PegEPO without a decline in hemoglobin levels could be achieved by administering PegEPO every two weeks, but not every four weeks (68). Additionally, it also provides frequent hemoglobin monitoring in the initial phase of switching. There have also been studies conducted to evaluate effects of weekly and biweekly intravenous PegEPO administration on erythropoiesis, showing that weekly administration is well tolerated as biweekly in HD patients (69). However, the conversion should be considered taking into account the factors related to the patient and the stage of CKD. The KDIGO recommendation on dosing of Pegylated erythropoietin is mentioned (**Table 2**).

**Table 2:** KDIGO recommended dosing for Pegylated Erythropoietin (6).

	Initial dose	Dose adjustment
Methoxy polyethylene glycol–epoetin beta	CKD not receiving dialysis: 0.6 μg/kg or 50–120 μg every 2 wk, or 1.5 μg/kg or 120–200 μg every month	CKD not receiving dialysis: Increase or decrease the dose and/or dosing frequency as needed (generally not given more than once every 2 wk)
	CKD G5D: 0.6 μg/kg every 2 wk (may round to a convenient dose)	CKD G5D: Increase the dose by 30–50 μg/dose if Hb rise is <1.0 g/dl (<10 g/l) in 4 wk. Decrease the dose by 30–50 μg/dose if Hb rise is >2 g/dl (>20 g/l) in 4 wk

It is a standard practice that ESA should be administered at the end of the dialysis session, and the same holds for PegEPO as well (70). Kawai et al. demonstrated that shortening the PegEPO treatment interval combined with iron supplementation may lead to the more efficient treatment of HD patients with iron deficiency (69). The panel agreed that shortening the dosing interval compared to the dose often allows for more gradual and predictable control over the hemoglobin level, helping to keep it within the desired target range. Additionally, it avoids the patient’s load to purchase a new dose every time.

To avoid underdosing, the panel agreed that to avoid confusions related to body weight-based dosing, the nearest upper µg strength can be prescribed considering its predictable pharmacokinetics and slow and steady rise in hemoglobin. As mentioned earlier also, initially the dose conversion may seem high, but studies have shown that total dose requirements of PegEPO come down overtime; therefore, it is not harmful to start with a slightly higher dose (70).

### 3.6 Topic: safety, outcomes, and cost

30. If cost is not a concern, PegEPO can be preferred over other ESAs considering its advantages like less frequency of administration, less hemoglobin variability and stable rise in hemoglobin [D].

31. PegEPO has a comparable safety profile to other ESAs [A].

32. PegEPO is cost-effective compared to more frequently used thrice weekly ESA injections [B].

33. PegEPO is safe to use in the elderly CKD population [B].

34. PegEPO is safe in patients with previous minor ESA-related adverse events [C].

35. Considering the local geographical and practical challenges, PegEPO seems to be a better option than other long and short-acting ESAs in Indian healthcare settings [D].

Rationale: In developing countries like India, cost of therapy remains a big concern for the treating physician as it might affect the adherence of the patients (71). In a study conducted in maintenance hemodialysis patients, PegEPO showed a probability of 0.60 to be cost-saving and 0.99 of probability of being cost-effective (72). In another study in non-dialysis patients, in-clinic ESA administration was associated with high excess annual costs, varying from USD 2,572 per patient for monthly dosing to USD 20,948 per patient for thrice-weekly dosing (73). ESA administration incurs both direct and indirect costs, including preparation and administration of each dose, as well as other supplies and consumables needed for the application of each drug. When PegEPO administration was compared with thrice weekly rHuEPO administration, there was a trend to reduced total active HCP time when comparing PegEPO vs epoetin alfa administration (74). When extrapolated to a full year of ESA therapy (156 administrations for epoetin alfa versus 12 administrations for PegEPO), the estimated staff costs were USD 82.68 (95% CI: 81.12–84.24) for epoetin alfa and USD 4.44 (95% CI: 4.08–4.80) for PegEPO, respectively (74). Therefore, in the calculation of the cost of therapy, indirect costs should also be calculated, including transportation, injection and other healthcare.

In ESAs, one of the most common adverse effects is injection site reactions. Adherence to subcutaneously administered therapies may be adversely affected by frequent injections and injection-site reactions, such as pain. Decreasing the frequency of administration improves adherence. Additionally, reduced frequency also contributes to fewer hemoglobin excursions. Therefore, PegEPO in fortnightly/monthly administration is a fair choice for patients taking s.c. injections.

It is important to note that, although ESA dosing has evolved from frequent administration to longer-acting formulations, safety concerns remain central. Higher ESA doses and attempts to achieve near-normal hemoglobin (Hb) levels have been associated with increased risks, including hypertension, thromboembolic events such as stroke, and vascular access thrombosis, particularly in dialysis patients. Hb overshoot—especially beyond 13 g/dl—further amplifies these risks. Patients with ESA hyporesponsiveness often require higher doses and experience disproportionate adverse events, suggesting dose-related toxicity. Additionally, concerns have been raised regarding potential tumour progression or recurrence with high ESA exposure. Collectively, these findings have led to more conservative Hb targets and cautious ESA use, emphasizing avoidance of high doses and rapid Hb correction to mitigate cardiovascular and thrombotic complications (75).

India is a vast country and there are multiple challenges concerning ESAs primarily concerning logistics and infrastructure constraints, especially in rural and remote areas. Issues like cold chain management related to challenges like temperature fluctuations, inefficient routes, high operational costs, regulatory compliance issues and price constitute an important part to maintain the compliance to the therapy (76). ESAs also require strict cold-chain storage to maintain stability and minimize the risk of immunogenicity, which may limit access in certain regions (77, 78). PegEPO advantages like fortnightly or monthly injections and comparable cost improve patient adherence. Additionally, it also reduces the burden on healthcare staff by reducing the workload in dialysis centers.

## 4 Conclusion

For both DD and NDD patients with CKD, this consensus offers helpful recommendations for maximizing the use of PegEPO for anemia management. It helps nephrologists expand safe and effective treatment options beyond traditional ESAs by incorporating expert insights and current evidence. However, these insights are largely based on the expert opinions and clinical experience of participating nephrologists, underscoring the need for additional region-specific research to strengthen clinical confidence and inform practice in real-world settings. These observations warrant validation through well-designed clinical trials and real-world studies. Nonetheless, they provide valuable preliminary guidance to treating nephrologists on various aspects of Peg-EPO use, including patient selection, dosing strategies, safety, and cost considerations.
