# Supplementary material for: Real-world use of pegylated erythropoietin in CKD-related anemia: an Indian modified Delphi consensus
Source: Front Nephrol. 2026 Sep 8;6:1864394. doi: 10.3389/fneph.2026.1864394 (PMC13595928; doi:10.3389/fneph.2026.1864394)
Supplement: Supplementary file 1 [file DataSheet1.pdf]

Round 1

Short acting ESAs are currently first choice of ESA therapy in Dialysis Dependent patients	50
Long acting ESAs are currently 1st choice for NDD patients	58.3
Pegylated EPO can be considered the first-line ESA in CKD stage 5D patients	66.7
Pegylated EPO can be considered the first-line ESA in CKD stage 3-5ND patients	100
The preferred starting dose of PegEPO in ESA-naive CKD patients should be weight-based (0.6 µg/kg IV/SC every 2 weeks)	66.7
Patients with Hb variability or frequent dose changes on darbepoetin or epoetin should be switched to Pegylated EPO	58.3
Conversion ratios (e.g., darbepoetin to CERA) are well established and practical in real-world settings	66.7
Pegylated EPO is suitable for switch patients from short acting ESAs	75
PegEPO provides better Hb stabilization compared to shorter-acting ESAs	75
Once-monthly dosing improves adherence and clinic workflow	91.7
Twice monthly dosing is better than monthly dosing in Dialysis dependent patients	66.7
Hb should be monitored every 2 weeks after initiation/dose adjustment with PegEPO	83.3
Shortening dosing interval preferable to increasing dose in Hb below target	54.6
IV PegEPO should be administered at end of dialysis session	83.3
IV iron should be administered proactively in DD patients on PegEPO	66.7
Ferritin >200 & TSAT >20% before initiating PegEPO	100
Oral iron adequate in most NDD CKD on PegEPO	54.6
Switch to PegEPO in patients with Hb variability/frequent dose changes	66.7
Switch to PegEPO in patients missing doses due to compliance	66.7
Target Hb should be maintained between 10–12 g/dL	100
Monthly Hb monitoring is sufficient after dose stabilization	100
More aggressive Hb correction increases thromboembolic risks	83.3
PegEPO has comparable safety profile to other ESAs	83.3
PegEPO is cost-effective when used monthly	75
Use of PegEPO reduces staff time and clinic burden in dialysis	91.7
PegEPO is safe to use in elderly CKD patients	66.67
Longer dosing intervals reduce Hb variability–associated adverse outcomes	66.67
PegEPO improves long-term outcomes vs shorter-acting ESAs	41
Improved Hb stability with PegEPO improves QoL	66.7
PegEPO is better suited than other ESAs considering local challenges	66.7
If cost is not a concern, PegEPO is preferable due to advantages	58.8

Round 2

Short acting EPOs are currently first choice of ESA therapy in DD patients	33.3
Long acting (DPO) are currently 1st choice for NDD patients	83.3
De Novo patients on ESAs are more suitable for PegEPO therapy	75
Patients who need ESA conversion from IV to SC route can be considered for PegEPO therapy	91.6
ESA hyporesponsive patients can be given a trial with PegEPO therapy	83.3
Patients who frequently misses their ESA injections and have poor compliance can be shifted to PegEPO therapy	100
The starting dose of PegEPO should be 50 mcg in denovo/switch patients	75
The starting dose of PegEPO should be 100 mcg in denovo/switch patients	25
The dose titration is based on Hb levels measured in one month	66.7
PegEPO provides better Hb stabilization compared to shorter-acting ESAs	100
Twice monthly dosing is better than monthly dosing in Dialysis dependent patients	91.7
For patients with Hb below target despite PegEPO, shortening the dosing interval is preferable to increasing the dose	66.6
In hemodialysis patients, PegEPO should be administered IV at the end of the dialysis session.	66.6
IV iron should be administered proactively in all DD patients initiated on Pegylated EPO	91.7
Oral iron supplementation is adequate in most non-dialysis CKD patients on PegEPO	58.3
Patients with Hb variability or frequent dose changes on darbepoetin or epoetin should be switched to Pegylated EPO	100
Switching to Pegylated EPO should be considered in patients missing ESA doses due to compliance issues	100
Conversion ratios (e.g., darbepoetin to PegEPO) are well established and practical in real-world settings	91.6
Pegylated EPO is cost-effective compared to more frequently used thrice weekly shorter-acting ESAs	83.3
Pegylated EPO is safe to use in elderly CKD patients without dose adjustments beyond standard recommendations	100
Considering the local geographical and practical challenges, Pegylated EPO seems to be a better option than other long and short acting ESAs	100

Round 3

Short acting EPO is currently the first choice for DD patients	50
Starting dose of PegEPO in denovo/switch patients should be 50 mcg fortnightly	83.3
Loading dose of 100 mcg can be considered while starting PegEPO in CKD anemia patients	83.3
Dose can be titrated measuring monthly Hb level	100
For patients not achieving the desired Hb level, shortening the dosing interval is preferable than changing the dose	100
In HD patients, PegEPO should be administered at the end of Dialysis session	100
Oral iron is sufficient with PegEPO in most NDD patients	33.3
More frequent Hb monitoring (every 2 weeks) is required during the first two months after switching to PegEPO	83.3
PegEPO is able to maintain Hb levels when administered once every 2-4 weeks SC or IV, in patients who were previously maintained on EPO alpha or Darbepoetin	100
Patients with stabilized Hb on fortnightly ESAs can be considered for safely switching to monthly PegEPO without compromising anemia control	100
Combining PegEPO with IV iron during the same visit improves patient compliance and outcomes	91.7
In case of dose discrepancy on per kg body weight, the nearest upper mcg dose can be considered for administration	100
Patients with stable iron stores (TSAT >25% and ferritin>200 ng/mL) are more likely to have predictable response to PegEPO	100
PegEPO is safe in patients with previous minor ESA-related adverse events	100

Statements which did not reach consensus

- Short acting EPO is currently the first choice for DD patients
- Oral iron is sufficient with PegEPO in most NDD patients

Abbreviations:

ESA: Erythropoeisis stimulating agents; PegEPO: Pegylated Erythropoeitin; CKD: Chronic Kidney Disease; DD: Dialysis Dependent; NDD: Non Dialysis Dependent; TSAT: Transferrin Saturation; SC: Subcutaneous; IV: Intravenous; Hb: Hemoglobin
