# Supplementary material for: Real-world use of pegylated erythropoietin in CKD-related anemia: an Indian modified Delphi consensus
Source: Front Nephrol. 2026 Sep 8;6:1864394. doi: 10.3389/fneph.2026.1864394 (PMC13595928; doi:10.3389/fneph.2026.1864394)
Supplement: Supplementary file 2 [file DataSheet2.pdf]

Grading of Statements:

Level A: Systemic Review/ Meta-analysis/ Randomised trials

Level B: Prospective/ Retrospective Observational evidence/ Registries/ Real World Evidence (Level B)

Level C: National or International Guidelines/Position statements/Expert recommendations

Level D: Expert opinion/ Pharmacoeconomic considerations/clinical practice-based insights where published evidence is limited

S.N	*1st statement*	*Final Statement*	*Evidence Grade*	*Evidence Type*	*Final Agreement*
1	Long-acting ESA is the first choice as ESA for anemia management in NDD patients	Long-acting ESA are first choice for anemia management in NDD patients	C	Guideline recommendations (KDIGO, clinical practice)	100%
2	De novo patients are more suitable for PegEPO therapy	De novo patients with renal anemia can be initiated on ESA therapy using PegEPO	B	Observational studies / real-world evidence	83%
3	PegEPO is suitable to switch patients from short acting ESAs	PegEPO is suitable to switch patients from short-acting ESAs	B	RCTs + extension studies	83%
4	PegEPO can be considered the first-line ESA in CKD stage 5D patients	PegEPO can be considered the first-line ESA in CKD stage 5D patients	B	Observational + limited comparative trials	100%
5	Patients who need ESA conversion from intravenous to subcutaneous are more suitable for PegEPO therapy	Patients who need ESA conversion from intravenous to subcutaneous can be shifted to PegEPO	C	Expert opinion / practice-based evidence	92%
6	PegEPO is safe to use in elderly CKD patients without dose adjustments beyond standard recommendation	PegEPO is safe to use in elderly CKD patients without dose adjustments beyond standard recommendation	B	Subgroup analyses + observational data	83%
7	ESA hyporesponsive patients can be prescribed PegEPO as ESA therapy	ESA hyporesponsive patients can be given a trial of PegEPO as ESA therapy after ruling out all the possible causes of ESA resistance	C	Expert consensus (limited direct trial evidence)	83%
8	Patients who frequently miss their ESA injections and have poor compliance can be shifted to PegEPO	Patients who frequently miss their ESA injections and have poor compliance can be shifted to PegEPO	B	Real-world adherence studies	100%
9	PegEPO provides better hemoglobin	PegEPO provides better hemoglobin	A	RCTs demonstrating Hb stability	100%

	stabilization compared to short acting ESAs	stabilization compared to short acting ESAs			
10	Ferritin >200 ng/mL and TSAT >20% should be ensured before initiating PegEPO	Ferritin >200 ng/mL and TSAT >20% should be ensured before initiating PegEPO	C	Guideline recommendations (KDIGO) + expert consensus	92%
11	Intravenous iron should be administered proactively in all DD patients to be initiated on PegEPO	Intravenous iron should be administered proactively in all DD patients to be initiated on PegEPO	C	Randomized trials (e.g., PIVOTAL study)	92%
12	Patients with stable iron stores (TSAT ≥25% and ferritin ≥200 ng/mL) are more likely to have predictable response to PegEPO	Patients with stable iron stores (TSAT ≥25% and ferritin ≥200 ng/mL) are more likely to have a predictable response to PegEPO	B	Observational studies / real-world evidence	100%
13	Combining PegEPO with intravenous iron during the same visit improves patient compliance and outcomes	Combining PegEPO with intravenous iron during the same visit might improve patient compliance and outcomes	B	Observational studies + practice-based evidence	83%
14	A once-monthly dosing frequency improves patient adherence and clinic workflow	A once-monthly dosing frequency improves patient adherence and clinic workflow	B	Observational studies + pharmacoeconomic / real-world practice data	83%
15	Longer dosing intervals with PegEPO reduce the risk of hemoglobin variability associated adverse outcomes	Longer dosing intervals with PegEPO reduce the risk of hemoglobin variability-associated adverse outcomes	B	Observational studies + post-hoc analyses	83%
16	Patients with hemoglobin variability or requiring frequent dose changes on Darbepoetin/ short acting ESAs should be shifted to PegEPO	Patients with hemoglobin variability or requiring frequent dose changes on Darbepoetin/ short acting ESAs can be shifted to PegEPO	B	Observational studies + clinical practice evidence	100%
17	Patients with stabilized hemoglobin on fortnightly ESA injections can be considered for safely switching to monthly PegEPO without compromising anemia control	Patients with stabilized hemoglobin on fortnightly ESA injections can be considered for safely switching to monthly PegEPO without compromising anemia control	A	Randomized controlled trials + extension studies	100%
18	PegEPO is able to maintain hemoglobin levels when administered once every 2-4 weeks, subcutaneous or	PegEPO can maintain hemoglobin levels when administered once every 2-4 weeks, subcutaneous or intravenous, in patients	A	Randomized trials (CERA studies) + comparative trials	92%

	intravenous, in patients who were previously maintained on EPO alpha or Darbepoetin	who were previously maintained on EPO alpha or Darbepoetin			
19	Hemoglobin should be monitored every 2 weeks after initiation or dose adjustment with PegEPO	Hemoglobin should be monitored every 2 weeks after initiation or dose adjustment with PegEPO	C	Guideline recommendations (KDIGO) + expert consensus	83%
20	Monthly hemoglobin monitoring is sufficient after dose stabilization	Monthly hemoglobin monitoring is sufficient after dose stabilization	C	Guideline recommendations + clinical practice standards	100%
21	More frequent hemoglobin monitoring is required during the first two months after switching to PegEPO	More frequent hemoglobin monitoring is required during the first two months after switching to PegEPO	C	Expert consensus + practice-based evidence	83%
22	Target hemoglobin should be maintained between 10-12 g/dL in patients on PegEPO	Target hemoglobin should be maintained between 10-12 g/dL in patients on PegEPO	C	Randomized trials + guideline recommendations (Indian, UKKA)	92%
23	The starting dose of PegEPO in de novo/ switch patients should be 50 µg fortnightly	The starting dose of PegEPO in de novo/ switch patients should be 50 µg fortnightly	C	KDIGO guideline recommendation	92
24	A loading dose of 100 µg can be considered in patients having hemoglobin <9 g/dL	A loading dose of 100 µg can be considered in patients having hemoglobin <9 g/dl	D	Practice based recommendation	83
25	Fortnightly dosing is better than monthly dosing	Fortnightly dosing is better than monthly dosing	C	Observational studies + subgroup analyses	92
26	Conversion ratios to PegEPO are well established and practical in real-world settings	Conversion ratios to PegEPO are well established and practical in real-world settings	A	Randomized trials + conversion studies	83
27	In hemodialysis patients, PegEPO should be administered at the end of dialysis session	In hemodialysis patients, PegEPO should be administered at the end of the dialysis session	C	Guideline recommendations + expert consensus	100
28	For patients not achieving the desired hemoglobin level, shortening the dosing interval is preferable than changing the dose	For patients not achieving the desired hemoglobin level, shortening the dosing interval is preferable to changing the dose	D	Practice based recommendation	92
29	In case of dose discrepancy on per kg body weight, the nearest upper µg dose can be considered for administration	In case of dose discrepancy on per kg body weight, the nearest upper µg dose can be considered for administration	C	Expert consensus	92
30	If cost is not a concern, PegEPO can be preferred over other ESAs considering its	If cost is not a concern, PegEPO can be preferred over other ESAs considering its	D	Practice based recommendation	83

	advantages like less frequency of administration, less hemoglobin variability and stable rise in hemoglobin	advantages like less frequency of administration, less hemoglobin variability and stable rise in hemoglobin			
31	PegEPO has a comparable safety profile to other ESAs	PegEPO has a comparable safety profile to other ESAs	A	Randomized controlled trials + meta-analyses	100
32	PegEPO is cost-effective compared to more frequently used thrice weekly ESA injections	PegEPO is cost-effective compared to more frequently used thrice weekly ESA injections	B	Pharmacoeconomic analyses + real-world data	83
33	PegEPO is safe to use in elderly CKD population	PegEPO is safe to use in the elderly CKD population	B	Observational studies + subgroup analyses	100
34	PegEPO is safe in patients with previous minor ESA-related adverse events	PegEPO is safe in patients with previous minor ESA-related adverse events	C	Expert opinion + limited observational evidence	100
35	Considering the local geographical and practical challenges, PegEPO seems to be a better option than other long and short acting ESAs in Indian healthcare settings	Considering the local geographical and practical challenges, PegEPO seems to be a better option than other long and short-acting ESAs in Indian healthcare settings	D	Practice-based recommendation	92

Statements which did not reach consensus

- Short acting EPO is currently the first choice for DD patients
- Oral iron is sufficient with PegEPO in most NDD patients

Abbreviations:

ESA: Erythropoeisis stimulating agents; PegEPO: Pegylated Erythropoeitin; CKD: Chronic Kidney Disease; DD: Dialysis Dependent; NDD: Non Dialysis Dependent; TSAT: Transferrin Saturation; SC: Subcutaneous; IV: Intravenous; Hb: Hemoglobin
